# Cytosine Base Editor-Mediated Multiplex Genome Editing to Accelerate Discovery of Novel Antibiotics in *Bacillus subtilis* and *Paenibacillus polymyxa*

**DOI:** 10.3389/fmicb.2021.691839

**Published:** 2021-05-28

**Authors:** Man Su Kim, Ha-Rim Kim, Da-Eun Jeong, Soo-Keun Choi

**Affiliations:** ^1^Infectious Disease Research Center, Korea Research Institute of Bioscience and Biotechnology (KRIBB), Daejeon, South Korea; ^2^Department of Biosystems and Bioengineering, KRIBB School of Biotechnology, University of Science and Technology (UST), Daejeon, South Korea

**Keywords:** base editor, multiplex genome editing, *Bacillus subtilis*, *Paenibacillus polymyxa*, antibiotics

## Abstract

Genome-based identification of new antibiotics is emerging as an alternative to traditional methods. However, uncovering hidden antibiotics under the background of known antibiotics remains a challenge. To over this problem using a quick and effective genetic approach, we developed a multiplex genome editing system using a cytosine base editor (CBE). The CBE system achieved simultaneous double, triple, quadruple, and quintuple gene editing with efficiencies of 100, 100, 83, and 75%, respectively, as well as the 100% editing efficiency of single targets in *Bacillus subtilis*. Whole-genome sequencing of the edited strains showed that they had an average of 8.5 off-target single-nucleotide variants at gRNA-independent positions. The CBE system was used to simultaneously knockout five known antibiotic biosynthetic gene clusters to leave only an uncharacterized polyketide biosynthetic gene cluster in *Paenibacillus polymyxa* E681. The polyketide showed antimicrobial activities against gram-positive bacteria, but not gram-negative bacteria and fungi. Therefore, our findings suggested that the CBE system might serve as a powerful tool for multiplex genome editing and greatly accelerating the unraveling of hidden antibiotics in *Bacillus* and *Paenibacillus* species.

## Introduction

The emergence and prevalence of multidrug-resistant bacteria (MDR) are a global health threat that causes ~700,000 deaths every year (Kollef et al., 2017). As resistance to most antibiotics is common, the availability of antibiotics for the treatment of severe bacterial infections is being depleted (Alanis, 2005; Frieri et al., 2017). Since the golden era of antibiotic discovery when nearly all antibiotics in use today were identified, the development of new antibiotics is gradually decreasing due to technical difficulties as well as unprofitability of developing new antibiotics and rapid acquisition of antibiotic resistance (Lewis, 2013; Brown and Wright, 2016). The Infectious Diseases Society of America (IDSA) has encouraged the development of antibiotics to counter this decline *via* the 10 × ‘20 Initiative; however, the majority of recently approved antibiotics have been modifications of existing chemical classes of antibiotics, rather than new chemical classes (Talbot et al., 2019). Therefore, the discovery of new classes of antibiotics is required to confront the spread of MDR and save patients’ lives.

The “Waksman platform” for screening soil-derived streptomycetes with antimicrobial activity was the largest successful discovery platform that helped to produce the major classes of antibiotics in the golden era of antibiotic discovery (Lewis, 2013). However, the platform was abandoned due to the rediscovery of known compounds, making it difficult to discover new antibiotics (Lewis, 2013). Since then, the field of antibiotic discovery has focused on developing synthetic antibiotics based on new platforms for high-throughput screening (HTS) and rational drug design. From 1995 to 2001, there have been over 70 HTS campaigns (Payne et al., 2007); however, these have limitations because it is difficult to identify compounds that can sufficiently penetrate the bacterial cell wall and have a reasonable antimicrobial spectrum (Lewis, 2013). Despite recent research to discover untapped sources of microbes in previously inaccessible or underexplored environments, for *Streptomyces* species, the presence of an enormous background of old known compounds remains one of the biggest barriers to the discovery of new antibiotics (Lewis, 2013; Hutchings et al., 2019). A process termed “dereplication” for the elimination of known antibiotics in microbial extracts has been used to resolve the issue of frequent re-isolation of the same known compound in antibiotic discovery. However, it is a laborious and resource-intensive process (Corley and Durley, 1994). Recently, dereplication has involved the use of analytical methods such as mass spectrometry, NMR-based metabolomics, and transcription profiling. However, these methods often require pure fractionations and are not suitable for initial screening approaches (Gaudêncio and Pereira, 2015; Genilloud, 2019). Therefore, a new platform not requiring a complex purification process is warranted for rapid dereplication in microbial strains producing multiple antibiotics for efficient screening and analysis of masked compounds.

Recently, advances in high throughput genome sequencing have confirmed that there are unexploited antibiotic biosynthetic gene clusters (BGCs) in diverse microbial sources, which may provide opportunities for the discovery of new antibiotics (Doroghazi et al., 2014; Genilloud, 2019). Concomitant with the accumulation of microbial genome sequences, *in silico* genome mining strategies have been developed, which may facilitate genome-based new antibiotic discovery (Weber and Kim, 2016; Lee et al., 2020). To date, several large-scale genome mining projects have focused on Actinobacteria, which have been an important source in the discovery of new antibiotics including β-lactams, rifamycins, aminoglycosides, tetracyclines, macrolides, and glycopeptides (Doroghazi et al., 2014; Adam et al., 2018; Chevrette et al., 2019). Additionally, bacterial species from the order Bacillales have significant potential for the discovery of new antibiotics, including antimicrobial peptides such as bacteriocin, lantibiotics, lipopeptides, and biosurfactants (Aleti et al., 2015; Zhao and Kuipers, 2016; Grubbs et al., 2017). However, various species from the order Bacillales, such as *Bacillus* and *Paenibacillus*, have remained underexplored in the field of antibiotics discovery, despite the distribution and diversity of BGCs that are different from that in Actinobacteria.

Many microorganisms have multiple BGCs, and *Bacillus* species have an average of 11 BGCs per strain (Grubbs et al., 2017), which poses an obstacle for the identification and analysis of a single antibiotic compound. Although a heterologous expression approach has been utilized in a clean host, there are several limitations such as the difficulty of large BGC cloning, incompatible regulatory systems, low production titers, and a lack of critical biosynthetic precursors or enzyme functions. To overcome these limitations and the time-consuming processes involved, new platforms that utilize the original strain are needed. Since a genetic approach could represent the best way for direct dereplication in the original strain, an efficient multiplex genome editing tool is required. Many genome editing approaches have been developed for microorganism (Suzuki et al., 2005; Karstentischer et al., 2006; Jeong et al., 2015), and currently the most valued technology is CRISPR/Cas-mediated homologous recombination, which has high accuracy and efficiency (So et al., 2017; Tian et al., 2017). Nonetheless, multiplex genome editing using the CRISPR/Cas systems remains to struggle owing to a low recombination efficiency of multiple double-strand breaks (DSBs) generated by Cas9 nucleases and the excess time required for iterative editing (Jiang et al., 2015; Adiego-Pérez et al., 2019; Lim and Choi, 2019). A recently developed tool called the cytosine base editor (CBE) involves the fusion of Cas9 nickase (nCas9; D10A mutation) and cytidine deaminase, which enables precise base editing in yeast or mammalian cells without DSBs (Komor et al., 2016, 2017). The CBE is highly valuable for multiplex genome editing due to its simplicity, high efficiency, high specificity, and low genome damage (Komor et al., 2016; Nishida et al., 2016); further, it does not require recombination, foreign DNA templates, or DSBs, unlike previous genome editing tools (Vento et al., 2019). Recently, a CBE system for *Bacillus subtilis* has been reported (Yu et al., 2020). However, the system had a narrow optimal editing window and the editing efficiency was significantly decreased when more than three targets were edited at the same time, indicating that the base editor for multiplex editing needs to be improved.

In this study, we developed a CBE-based highly efficient multi-gene editing system in *B. subtilis* and demonstrated that this system is a powerful genetic dereplication tool through the one-step inactivation of five known BGCs of *Paenibacillus polymyxa* E681 and subsequent characterization of a hidden polyketide antibiotic. We believe that this platform may greatly accelerate the discovery of hidden antibiotics *via* rapid genetic dereplication in *Bacillus* and *Paenibacillus* species.

## Materials and Methods

### Strains and Culture Conditions

The strains used in this study are listed in Supplementary Table S1. The *Escherichia coli* strains DH5α or MC1061 were used as the general cloning host and *E. coli* S17-1 (KCTC 2432) was used for conjugation. The *E. coli* and *B. subtilis* strains were grown in Luria-Bertani (LB; Affymetrix, Santa Clara, CA, United States) medium at 37°C. The *P. polymyxa* strains were grown in Tryptic soy broth (TSB; Difco, Detroit, MI, United States) or Tryptic soy agar (TSA) at 30°C. When required, the medium was supplemented with chloramphenicol (5 μg/ml for *B. subtilis* and 7.5 μg/ml for *P. polymyxa*), ampicillin (100 μg/ml), or polymyxin B (10 μg/ml). Transformation of *B. subtilis* and *P. polymyxa* was performed as reported previously (Richhardt et al., 2010; So et al., 2017). Indicator strains obtained from the American Type Culture Collection (ATCC), the Korean Collection for Type Cultures (KCTC), or the Korean Agricultural Culture Collection (KACC) for the antimicrobial activity assay were grown as follows: *Bacillus cereus* ATCC 4342™ was grown in LB broth or LB agar at 30°C. *E. coli* KCTC 22003 and *Acinetobacter baumannii* ATCC 19606™ were grown in LB broth or LB agar at 37°C. *Micrococcus luteus* KCTC 2177 was grown in TSB or TSA at 30°C. *Staphylococcus aureus* ATCC 25923™ and *Pseudomonas aeruginosa* ATCC 27853™ were grown in TSB or TSA at 37°C. *Fusarium graminearum* KACC 41040, *Fusarium solani* KCTC 6326, and *Rhizoctonia solani* KACC 40146 were grown in potato dextrose agar (PDA; Difco) at 25°C.

### Vector Construction

The plasmids and primers used in this study are listed in Supplementary Tables S2 and S3, respectively. The plasmid backbone for CBE mutagenesis was modified from the pAgR plasmid used previously (So et al., 2017). To introduce the P*_grac_* promoter (Phan et al., 2015) for CBE expression into the pAgR plasmid, the primers lacI-grac-F and lacI-grac-R, ter-F and ter-R were used to amplify P*_grac_* promoter and the terminator, respectively, using pHCas9 as a template (So et al., 2017). The two PCR products and SacI- and NsiI-digested pAgR were fused to construct the plasmid pAgR-Pgrac using the Cold Fusion Cloning kit (System Biosciences Inc., Palo Alto, CA, United States) according to the manufacturer’s instructions. The plasmid pAgR-Pspac was constructed similarly using the pMUTIN4 as a template to introduce the P*_spac_* promoter (Yansura and Henner, 1984) for CBE expression.

The plasmid pUC-dCas9 was constructed by the fusion of EcoRI/HindIII-digested pUC19 with *dCas9* fragments that were amplified from pScI_dCas9-CDA-UL (Addgene plasmid 108551) using the primers Cas9-F and Cas9-R. The rat cytidine deaminase (rAPOBEC1) gene was synthesized by Bioneer Co. (Daejeon, Republic of Korea; Supplementary Table S4). *rAPOBEC1* was amplified using primers APOBEC-F and APOBEC-R and was fused to AarI-digested pUC-dCas9 to construct pUC-APOBEC1-dCas9. Next, the pUC-APOBEC1-dCas9 was digested with BseRI and fused to a uracil glycosylase inhibitor (UGI) gene amplified from pScI_dCas9-CDA-UL using the primers UGI-F and UGI-R to construct pUC-CBE1. For the construction of pUC-CBE2, two UGIs were amplified from pScI_dCas9-CDA-UL using the primers UGI-F/UGI-R2 and UGI-F2/UGI-R, followed by fusion to the BseRI-digested pUC-APOBEC1-dCas9. To construct pUC-RBS-dCas9, AarI-digested pUC-dCas9 was ligated with an oligonucleotide generated by mixing the synthetic primers RBS-F and RBS-B. For pUC-CBE3, BseRI-digested pUC-RBS-dCas9 was fused to a *PmCDA1* (*Petromyzon marinus* cytidine deaminase 1)-*UGI*-LVA (protein degradation tag) fragment amplified from pScI_dCas9-CDA-UL using the primers CDA-UL-F and CDA-UL-R. For pUC-CBE4, BseRI-digested pUC-RBS-dCas9 was fused to the *PmCDA1*-*UGI* fragment amplified from pScI_dCas9-CDA-UL using the primers CDA-UL-F and CDA-UL-R2. The pUC-CBE plasmids were digested with FseI and SpeI and ligated with FseI- and SpeI-digested pAgR-Pgrac, to construct the plasmid pAgR-gCBE. The plasmid pAgR-sCBE4 was constructed similarly.

The multiplexing plasmid was modified from pAgR-Pspac to remove the extra Type IIS BsaI restriction sites and repetitive sequences that affect plasmid stability. To construct pMgR-Pspac, the primers mPAD-F1 and mPAD-R2, mPAD-F2 and mPAD-R3, mPAD-F3 and mPAD-R4, and mPAD-F4 and mPAD-R1 were used to amplify the four fragments from pAgR-Pspac, which were fused using the Cold Fusion Cloning kit. The sgRNA cassette of pAgR-Pspac was replaced with a green fluorescent protein (GFP)-dropout cassette flanked by two Type IIS BsaI restriction sites using the primers Egfp-BsaI-F and Egfp-BsaI-R from a plasmid containing P*_grac_*-*gfp* that was constructed in our lab similar to other literature (Phan et al., 2015). Next, the above vector was digested with FseI and SpeI and ligated with a similarly digested pUC-CBE4 to generate pMGold-sCBE4.

### gRNA Design and Cloning

There exist four codons (CAG: Gln, CAA: Gln, CGA: Arg, and TGG: Trp) that could be changed to stop codons by C-to-T conversion. The gRNAs with the four codons within the optimal editing window in our target genes were selected using the CRISPy-web site (Tong et al., 2019).^1^ The oligonucleotides containing the 20 bp gRNA generated by mixing synthetic primers (Supplementary Table S3) were ligated with the AarI-digested pAgR-derived plasmids. sgRNA synthesis was performed under the control of the P*_ara_* promoter as previously described (So et al., 2017). To construct pAgR-sCBE4-AN carrying two gRNAs, the primers BglII-sgRNA-F and BamClaHin-sgRNA-R were used to amplify the *nprE* targeting sgRNA cassette using pAgR-sCBE4-N as a template. The PCR product was digested with BglII and HindIII and ligated with the large fragment of BamHI and HindIII-digested pAgR-sCBE4-A to construct the plasmid pAgR-sCBE4-AN. Plasmids for triple, quadruple, and quintuple targets were also constructed using this stepwise cloning strategy. In constructing plasmids for the multiplex editing of antibiotic biosynthetic gene clusters of *P. polymyxa*, we applied the Golden-Gate assembly (Liao et al., 2019) as step-wise cloning is a time-consuming and laborious process. To construct pMgold-sCBE4-PFPTP (Supplementary Table S2), primer sets BB-vec-sgF/Bsa-sgR1, Bsa-sgF1/Bsa-sgR2, Bsa-sgF2/Bsa-sgR3, Bsa-sgF3/Bsa-sgR4, and Bsa-sgF4/SCBB-vec-sgR having the BsaI restriction enzyme sites (Supplementary Table S3) were used to amplify the *pnlB*, *fusA*, *phnC*, *triD*, and *pmxE*-targeting sgRNA cassettes, respectively. The protocol for the Golden-Gate assembly was modified from a previous publication (Liao et al., 2019). The reaction mixture contains 100 ng of each sgRNA cassette, 100 ng of the backbone plasmid (pMgold-sCBE4), 40 units of T4 DNA ligase (Promega Co., Madison, WI, United States), 30 units of BsaI-HFv2 (New England Biolabs Inc., Ipswich, MA, United States), and 2.5 μl of the T4 DNA ligation buffer. After adjusting the reaction volume to 25 μl with distilled water, a thermocycler was used to perform 25 cycles of digestion and ligation (37°C for 1 min and 16°C for 1 min) followed by a heat inactivation step (60°C for 5 min). The ligation mixture was used to transform *E. coli* DH5α cells.

### CBE-Mediated Mutagenesis

Transformations of *B. subtilis* 168 and *P. polymyxa* E681 were performed through the previously described natural competence (So et al., 2017) and conjugation methods (Richhardt et al., 2010), respectively. The CBE4 was expressed using the inducible promoters P*_grac_* or P*_spac_*, but a leaky expression without IPTG inducers was sufficient to induce the mutations. Randomly selected transformants were analyzed by DNA sequencing to confirm the mutations. Curing the CBE4 plasmids was conducted using a method described previously (So et al., 2017). The CBE4 plasmid backbone is the same as that for the previously reported plasmid pB0A (So et al., 2017) which showed a 50% curing efficiency. The plasmid-free antibiotic-sensitive colonies were reconfirmed for harboring the relevant mutations by DNA sequencing.

### Whole-Genome Sequencing of the Mutants

Genomic DNA was extracted from the *Bacillus* strains using the Wizard Genomic DNA Purification kit (Promega Co.) according to the manufacturer’s instructions. Library construction and genome sequencing were performed on the NovaSeq 6000 platform (Illumina Inc., San Diego, CA, United States) at Bioneer Co. For data analysis procedures, the reads obtained from Illumina sequencing were mapped to the *B. subtilis* 168 reference genome (Accession: NC_000964.3) using the Burrows-Wheeler Aligner (BWA, version 0.7.17; Li and Durbin, 2010), followed by PCR duplicate removal, variant calling, and annotation using the PicardTools (version 1.98), Genome Analysis Tool Kit (GATK) HaplotypeCaller (DePristo et al., 2011), and SnpEff tools (version 4.1; Cingolani et al., 2012), respectively. The output file was rearranged using Excel (Microsoft, Redmond, WA, United States). The sequence data were deposited to the National Center for Biotechnology Information (NCBI) under BioProject accession number PRJNA682711. The raw data are available in the Sequence Read Archive (SRA) under accession numbers SRR13203121–SRR13203130.

## Results

### Construction of a Highly-Efficient Base Editor System in *Bacillus subtilis*

**Figure 1:**
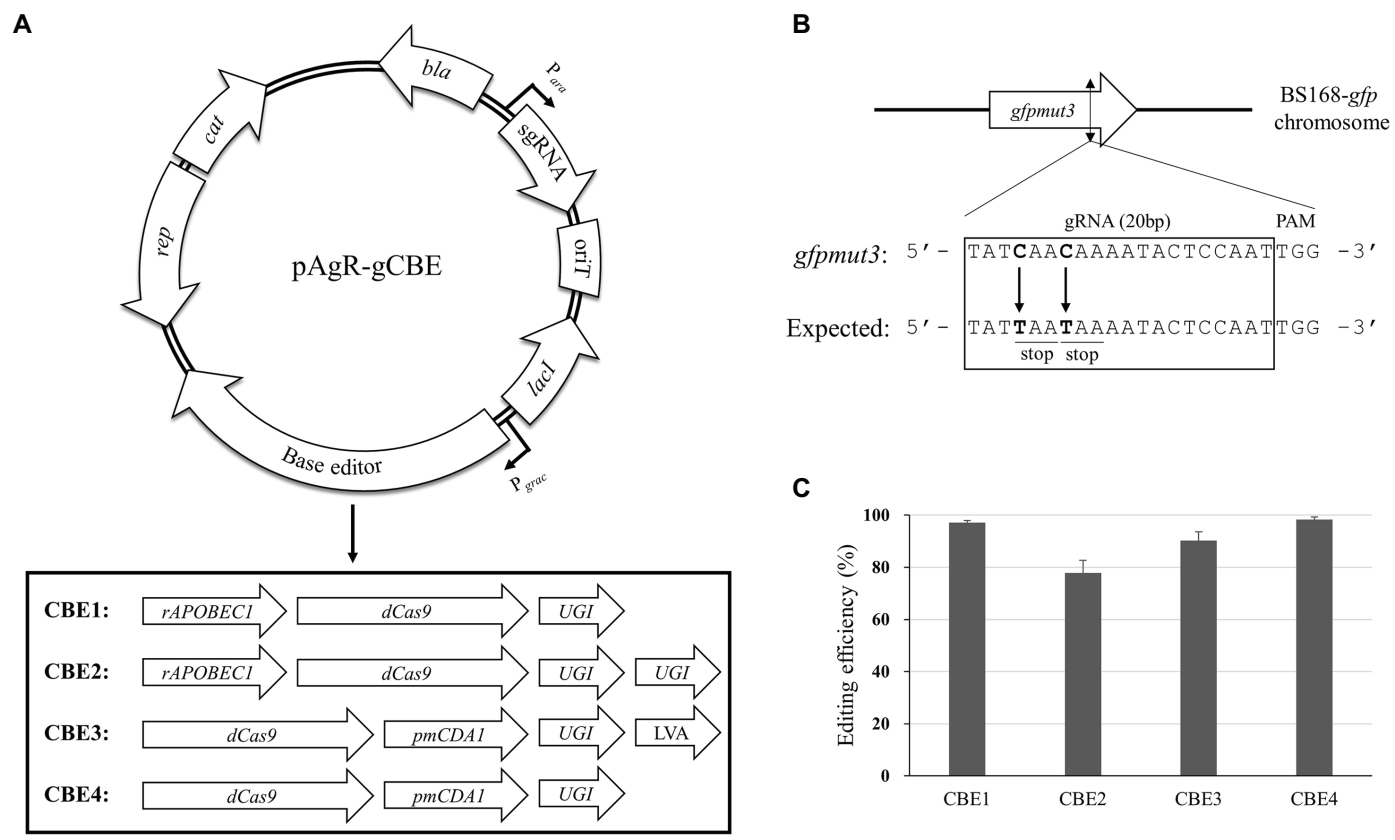
Cytosine base editor (CBE)-mediated mutagenesis in *Bacillus subtilis*. **(A)** Four types of CBE were constructed by the combination of a dCas9, two cytidine deaminases (rAPOBEC1 and PmCDA1), uracil glycosylase inhibitor (UGI), and a protein degradation tag (LVA). The CBE gene and sgRNA were under the control of the P*_grac_* and P*_ara_* promoters, respectively. **(B)** The chromosomal gRNA target site for introducing stop-codons into the *gfpmut3* gene of BS168-*gfp* strain. The gRNA-binding site is indicated by the black box including the target base (bold) and the expected codons (underline). **(C)** Editing efficiencies of the four CBEs. The efficiency was calculated using the proportion of green fluorescent protein (GFP)-negative transformants relative to the total number of transformants. The bars display the means of three independent experiments, with the error bars indicating SDs.

The base editor system contains an nCas9 fused with either cytidine deaminase or adenosine deaminase, which can induce C-to-T or A-to-G conversion, respectively. For *Bacillus* species, we selected the cytidine deaminase-containing system because it could create a stop codon within the target genes. Although the eukaryotic system contains nCas9, we used the catalytically inactive “dead” Cas9 (dCas9; D10A and H840A mutations) in this study because the *nCas9*-carrying plasmid showed a low transformation efficiency (Supplementary Figure S1A). The rat cytidine deaminase (rAPOBEC1) and *P. marinus* cytidine deaminase (PmCDA1) have been widely used as a component of CBE (Tang et al., 2019). To increase the efficiency of base editors, the addition of UGI to prevent the removal of uracil residues by base excision repair and a protein degradation tag (LVA tag) to reduce the cell burden has been reported (Banno et al., 2018). In this study, we designed four different constructs to test their efficiency as CBEs using rAPOBEC1, PmCDA1, UGI, LVA, and dCas9 (Figure 1A). According to the previous studies, the rAPOBEC1 and PmCDA1 are fused to the N-terminus and C-terminus of dCas9, respectively (Komor et al., 2017; Banno et al., 2018). To select the optimal base editor system, we applied the four constructs with *gfpmut3*-targeting sgRNA to introduce stop-codons within the *gfpmut3* gene of BS168-*gfp* (GFP expressing *B. subtilis* 168; Jeong et al., 2018; Figure 1B). We calculated the mutation efficiency of the four CBEs using the proportion of GFP-negative transformants among the total transformants. Accordingly, the CBE4 construct showed the highest efficiency (98.25%), followed by CBE1 (97.07%), CBE3 (90.17%), and CBE2 (77.85%; Figure 1C). Although the inducible promoter P*_grac_* was used to express CBEs, IPTG inducer was not added to induce mutations, suggesting that the leaky expression of the CBEs might be sufficient to induce high-efficiency mutations. Sequencing analysis revealed that all the GFP-negative transformants contained the expected C-to-T substitution at position 17 upstream of the protospacer-adjacent motif (PAM) of the *gfpmut3*-targeting sgRNA. The C-to-T substitution at position 14 occurred with a low efficiency in CBE1 and CBE4, but not in CBE2 and CBE3 systems, suggesting that position 14 might be out of the editing window of the CBE systems (Supplementary Figure S1B). As CBE4 showed the highest mutational efficiency among the four CBEs, it was used in further analyses.

### The Editing Window of CBE4

**Figure 2:**
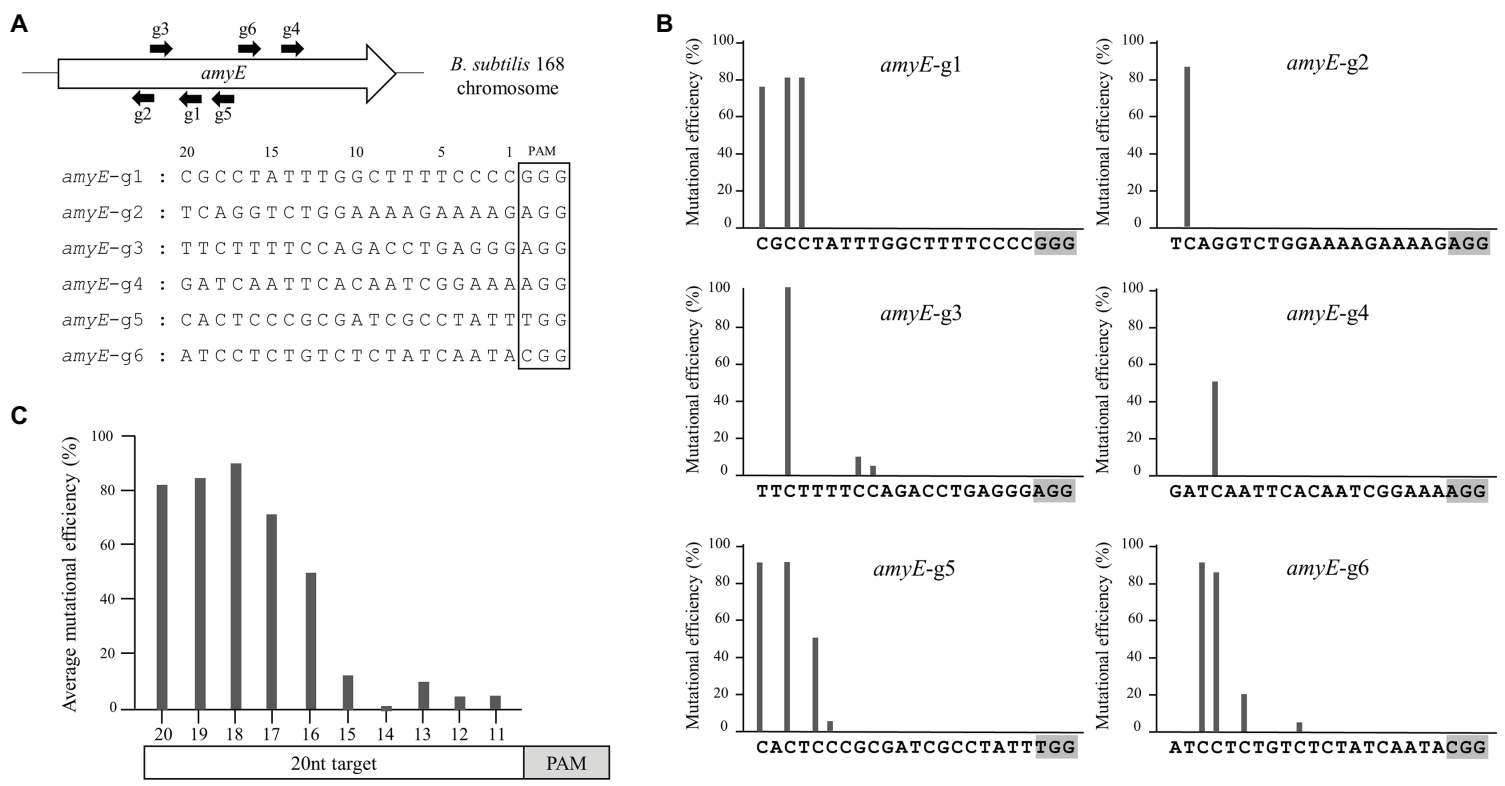
The editing window of CBE4. **(A)** Location and sequence of six gRNAs with different target positions in the *amyE* gene of *B. subtilis*. The numbers above the sequence indicate the nucleotide positions upstream of the protospacer-adjacent motif (PAM). **(B)** Mutation efficiencies of CBE4 for each gRNA. After randomly selecting 10 transformants per gRNA (60 transformants in total), the *amyE* mutations in these were analyzed by sequencing. The average efficiencies of two independent experiments are plotted. **(C)** The combined average mutation efficiency at each position for six targets.

**Figure 3:**
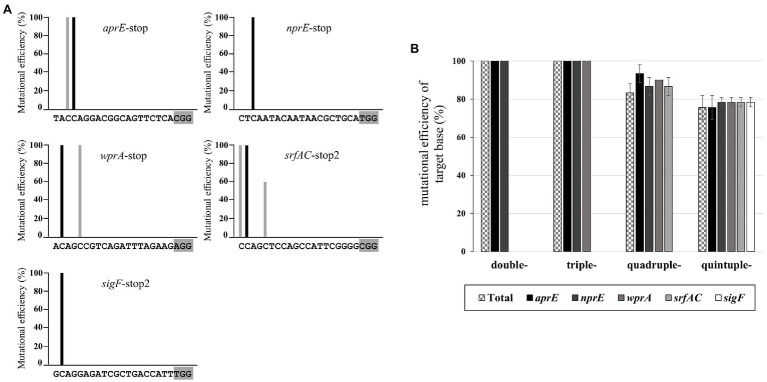
CBE4-mediated multiplex genome editing. **(A)** Mutation efficiency of single target genes. The nucleotide sequences show a 20 bp gRNA containing a gray shaded PAM motif. The mutation efficiency is indicated by the black bars for stop codon-generating targets and the gray bars for other targets not generating stop codons. The target genes from 10 randomly selected transformants per gRNA (50 transformants in total) were analyzed by sequencing, and the average mutation efficiencies of three independent experiments are plotted. **(B)** Mutation efficiency of multiplex editing for double, triple, quadruple, and quintuple targets by CBE4. “Total” (checkerboard bar) indicates the efficiency of simultaneous editing of the target genes. The target genes from 10 randomly selected transformants per vector (40 transformants in total) were analyzed by sequencing. The bars display the means of three independent experiments, with the error bars indicating SDs.

It is important to understand the editing window of CBE in designing gRNA with high efficiency at the desired position. To investigate the editing window of CBE4, we selected six gRNA binding sites in the *amyE* gene of *B. subtilis* 168 as editing targets, in which the cytosine bases were distributed across the 1–20 positions upstream of the PAMs (Figure 2A). We constructed six plasmids with different gRNAs targeting the *amyE* gene (Supplementary Table S2) and introduced them into BS5417 (*B. subtilis* 168 *thrC*::P*_xyl_*-*comK*). Ten transformants per gRNA (60 transformants in total) were randomly selected, and the mutations were analyzed by sequencing. The six targets showed highly efficient mutagenesis (50–100%) at positions 16–20, whereas positions 11–15 showed poor mutagenesis efficiencies (5–20%; Figure 2B). Despite the presence of cytosines between positions 1–10 of the six targets, C-to-T conversion was not observed. The average mutation frequency by CBE4 for the six targets was shown to be an optimal editing window at positions 16–20, and a high mutational efficiency of over 71% was observed at positions 17–20 (Figure 2C). Based on this window, the editing efficiency of CBE4 was confirmed for the *aprE*, *nprE*, *wprA*, *srfAC*, and *sigF* genes. The plasmids containing a single gRNA targeting each gene were constructed (Supplementary Table S2) and introduced into BS5417 to generate a stop codon within each gene by C-to-T conversion. Sequence analyses of the target genes showed that the cytosine bases at 17–20 position exhibited 100% mutation efficiency, excluding one target in *nprE* (position 20; Figure 3A). It is unclear why the cytosine at position 20 in *nprE* did not change, although a C-to-T conversion occurred at position 18 in *nprE*. In contrast, the two targets at position 20 in *amyE*-g1 and *amyE*-g5 (Figure 2B), and the target at position 20 in *srfAC*-stop2 (Figure 3A) changed successfully. Therefore, although the 20th position of *nprE*-stop was an exceptional case, the positions 17–20 from PAM may be prioritized in gRNA design for CBE4-meditated mutagenesis.

### Simultaneous Editing of Multiple Targets Using CBE4

Previously developed multiplex genome editing tools have shown decreased efficiency as the number of editing sites increased (Liu et al., 2019); therefore, we evaluated the multiplexing capacity of our base editor system. The vectors, pAgR-gCBE4-SS and pAgR-gCBE4-SSA (Supplementary Table S2), for simultaneous editing against double targets (*sigF* and *srfAC*) and triple targets (*sigF*, *srfAC*, and *aprE*), respectively, were constructed and introduced into BS5417. About 50% of the transformants for double targets showed *sigF* mutants with a transparent morphology. However, the *sigF* mutation phenotype was not observed in transformants for triple targets (Supplementary Figure S2). These results indicated that current system was insufficient to induce simultaneous editing of multiple targets, even without evaluating the editing efficiencies of *srfAC* and *aprE* targets. We assumed that the high expression level of CBE4 under P*_grac_* may increase the cell burden and affect the efficiency of simultaneous editing. It has been reported that the P*_grac_* promoter is 50 times stronger than P*_spac_* (Phan et al., 2015). Thus P*_grac_* was replaced with the P*_spac_* promoter to lower the expression level of CBE4. Using the changed system, four vectors (pAgR-sCBE4-AN, pAgR-sCBE4-ANW, pAgR-sCBE4-ANWS, and pAgR-sCBE4-ANWSF) for simultaneous editing of 2–5 targets were constructed (Supplementary Table S2) and introduced into BS5417. Sequencing analyses of the targets from 10 randomly selected transformants per vectors (40 transformants in total) revealed that the simultaneous editing efficiencies for double, triple, quadruple, and quintuple targets were 100, 100, 83.3, and 75.5%, respectively (Figure 3B). When transformants that were not simultaneously edited in the quadruple and quintuple targets were analyzed *via* PCR and sequencing, it was confirmed that the plasmid component including CBE4 was present, but there was a partial loss of the sgRNA cassette.

### Off-Target Mutations in CBE4-Edited Strains

**Figure 4:**
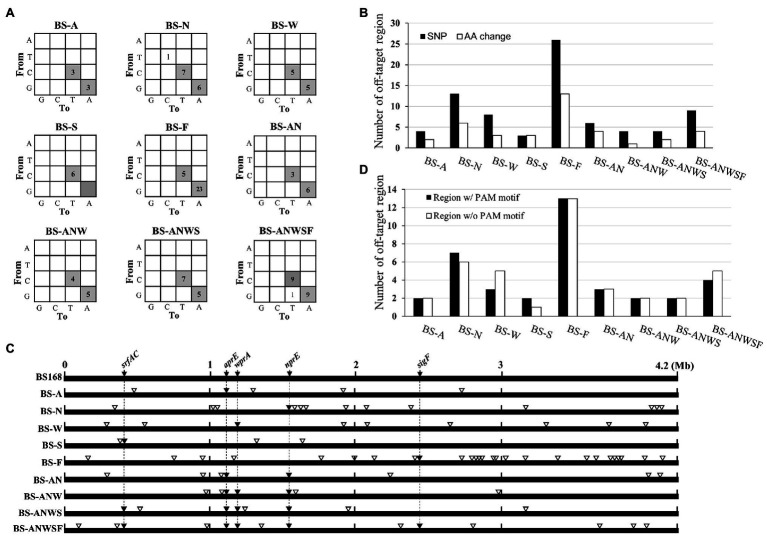
Genome-wide evaluation of off-target mutations in CEB4-edited cells. **(A)** The distribution of nucleotide changes in the genome of the mutant strains vs. the unedited control strain BS5417. The numbers in each cell indicate the number of nucleotide changes. C-to-T and G-to-A conversions that can be achieved by CBE4 are shown in gray. **(B)** The numbers of amino acid changes (white bar) caused by the nucleotide modifications (black bar) in the off-target sites. **(C)** Schematic location of off-targets in the mutant genomes. The location of on-target and off-targets are indicated by black inverted triangles (▼) and white inverted triangles (▽), respectively. **(D)** The number of presence (black bar) or absence (white bar) of the PAM motif at the off-target sites.

In genome editing, the off-target effect is an important issue. Although base editor has been reported to have an off-target effect (Liang and Huang, 2019), detailed analyses of the off-target mutations generated by multiplex genome editing have not been reported. To analyze the genome-wide off-target mutations generated in CBE4-edited strains, we performed whole-genome sequencing of 10 strains including single- (BS-A, BS-N, BS-W, BS-S, and BS-F), double- (BS-AN), triple- (BS-ANW), quadruple- (BS-ANWS), and quintuple- (BS-ANWSF) edited strains and an unedited control strain BS5417 (Supplementary Table S1). The results showed that the sequenced strains contained only single nucleotide variations (SNVs), but not other genetic variations such as deletions and insertions (Supplementary NGS data). Of the total 113 SNVs, C-to-T or G-to-A conversions were found to account for 98.2% of the mutations, indicating that the cytidine deaminase activity of CBE4 had a major effect on base editing (Figure 4A). The off-target sites showed an average of 8.5 SNVs including 4.2 amino acid changes per strain (Figure 4B). Interestingly, the off-target SNVs did not accumulate as the number of gRNAs increased. The off-target editing took place mostly in the single target BS-F, and the next most frequent was the single target BS-N (Figure 4B). Despite performing CBE4 mutagenesis using the same gRNA for each gene, there was no common off-target site between the multiplex and single edited strains (Figure 4C). When the total off-target sites were examined on the genome, it was confirmed that half of them did not have a significant PAM (Figure 4D). Even in the off-target sites where the PAM was present, the nucleotide similarity with each on-target gRNA was very low at ~25%.

### Application of CBE4 to Unveil an Uncharacterized Antibiotic in *P. polymyxa* E681

**Figure 5:**
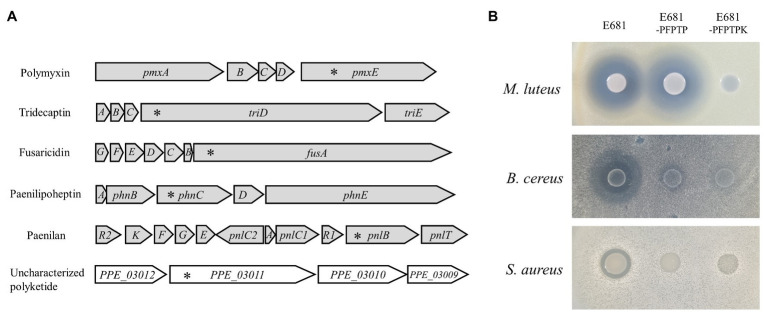
Application of CBE4 for antibiotic dereplication in *Paenibacillus polymyxa* E681. **(A)** Gene structure of the BGCs in *P. polymyxa* E681. The five BGCs represented in gray produce polymyxin, tridecaptin, fusaricidin, paenilipoheptin, and paenilan, respectively. The uncharacterized polyketide is represented in white. Asterisks indicate the mutation sites. **(B)** Antibacterial activities of *P. polymyxa* strains against the three Gram-positive bacteria, *Micrococcus luteus*, *Bacillus cereus*, and *Staphylococcus aureus*. Five known BGCs were knocked out using CBE4 in the strain E681-PFPTP leaving only the uncharacterized polyketide. In the E681-PFPTPK, all six BGCs were inactivated.

Multiplex genome editing tools can be applied to a wide variety of fields in research and industry, especially for the genome-based discovery of new antibiotics. Results from microbial genome sequencing have shown that many microorganisms have multiple BGCs. If there is a new BGC among multiple BGCs, the activity of the antibiotic it produces is masked by those of known compounds, which makes antibiotic characterization difficult. We selected *P. polymyxa* E681 as proof of concept to apply CBE4-based multiplex genome editing to unveil hidden antibiotics. There exist six BGCs in the *P. polymyxa* E681 genome (Jeong et al., 2019), where five of them are known to produce the antibiotics fusaricidin, paenilan, tridecaptin, paenilipoheptin, and polymyxin, whereas the sixth BGC produces a polyketide whose properties remain unknown (Figure 5A). To construct a strain that produces the uncharacterized polyketide alone, the plasmid pMgold-sCBE4-PFPTP (Supplementary Table S1) for a one-step disruption of the five known BGCs was introduced into *P. polymyxa* E681 through conjugation using *E. coli* S17-1 as a plasmid donor. Three transconjugants were selected, and the introduction of stop-codons into the *pnlB*, *fusA*, *phnC*, *trdA*, and *pmxE* genes of the transconjugants (E681-PFPTP) was analyzed by sequencing. Therefore, we confirmed that all target genes were simultaneously mutated at the desired positions in the three transconjugants. We established the strain E681-PFPTPK, wherein the remainder of the polyketide gene was disrupted using the plasmid pAgR-sCBE4-K (Supplementary Table S2), and used it as the control strain for antimicrobial assays. To evaluate the antimicrobial spectrum of the uncharacterized polyketide, we tested its antibacterial (*M. luteus*, *B. cereus*, and *S. aureus* for Gram-positive; *E. coli*, *P. aeruginosa*, and *A. baumannii* for Gram-negative) and antifungal activity (*F. solani*, *R. solani*, and *F. graminearum*). The polyketide produced from E681-PFPTP showed strong antibacterial activity against *M. luteus* and weak antibacterial activity against *B. cereus* among Gram-positive (Figure 5B), and no antibacterial activity against Gram-negative strains and fungi (Supplementary Figure S3). Similar to a previous study (Park et al., 2017), E681-PFPTPK with knockout mutations in all six BGCs showed no antimicrobial activity (Figure 5B). For further characterization, the polyketide was isolated and purified from the E681-PFPTP culture through solvent partitioning and column chromatography. As per the UV spectral data, the active fraction exhibited strong absorption at λ_max_ 299, 312, and 327 nm, indicating the presence of four conjugated double bonds in the typical UV spectrum for polyene (Supplementary Figure S4A). Further, we attempted to analyze the chemical structure of the polyketide, but the structural prediction was difficult because of the instability of the compound (Supplementary Figure S4B) as in the case of bacillaene that was reported previously (Moldenhauer et al., 2007). We believe that the polyketide may be a polyene compound due to its genetic similarity to the polyene antibiotic bacillaene, instability of compound, and the presence of conjugated double bonds observed in the UV spectrum.

## Discussion

Since the development of CBE system in eukaryotes (Komor et al., 2016), base editors have been gradually applied to prokaryotes such as *E. coli* (Banno et al., 2018), *P. aeruginosa* (Chen et al., 2018), *S. aureus* (Gu et al., 2018), *Streptomyces* (Tong et al., 2019), *Corynebacterium glutamicum* (Wang et al., 2018), and *B. subtilis* (Yu et al., 2020). While the eukaryotic CBE system contains nCas9, we used dCas9 as previously reported (Banno et al., 2018; Yu et al., 2020) because we found that nCas9 affects transformation efficiency in *B. subtilis* (Supplementary Figure S1A). For base editing, cytidine deaminases, rAPOBEC1, and PmCDA1 have been widely used as the component of CBEs. In this study, CBE1 and CBE4 containing rAPOBEC1 and PmCDA1, respectively, exhibited similar base editing efficiencies (Figure 1C). We chose the PmCDA1-based CBE (CBE4) for further analyses, as it showed a slightly higher efficiency than CBE1, nonetheless, the CBE1 could also be used as an efficient base editor in *B. subtilis*. The editing windows of the PmCDA1-based CBEs were similar to those reported in a previous study, where the editing window exists at positions 16–20 (Figure 2C; Wang et al., 2021). The CBEs used in this study contained a UGI to increase the mutation efficiency, as demonstrated previously (Komor et al., 2016). Another previous study showed that application of two UGIs provides better efficiency in inducing mutations than a single UGI in eukaryotes (Komor et al., 2017). Contrastingly, our study findings indicated that excess UGI had an adverse effect on *B. subtilis* (Figure 1C). It has been reported that the use of UGI in combination with a LVA tag results in a robust increase in the mutation efficiency of CBEs in *E. coli* (Banno et al., 2018). However, the UGI-LVA system (CBE3) in the present study displayed a somewhat reduced mutation efficiency compared with the system without the LVA tag (CBE4) in *B. subtilis*. Therefore, the dCas9-PmCDA1-UGI might be the optimum CBE system for base editing in *B. subtilis*. For most targets, CBE4-mediated mutagenesis appeared to follow the rules of the optimal editing window; however, an exceptional case was observed in the specific gRNA (position 20 of *nprE*-stop). This may be due to the gRNA secondary structure (Jensen et al., 2017) or the sequence preference of PmCDA1 (Lada et al., 2011) that affects the efficiency of CBE4.

Recently, the use of PmCDA1-mediated base editing has been reported in *B. subtilis* (Yu et al., 2020). The system showed high editing efficiency at position 18 from PAM alone. Position 17 showed approximately half the efficiency of position 18, and the remaining positions showed very low efficiency. In contrast, our system showed high editing efficiencies at positions 17–20, indicating that our system has a broad optimal editing window compared to the previous system. The narrow optimal editing window of the previous system may be caused by the lack of UGI. In the previous system, the efficiency was significantly reduced when more than three targets were edited simultaneously, whereas our system did not show significantly reduced efficiency. The previous system used a P*_grac_* promoter to express the base editor. In our study, the editing efficiency was significantly reduced when using the P*_grac_* promoter to express CBE. Instead, we achieved simultaneous multiplex editing with high efficiency by using a weak P*_spac_* promoter to express CBE. These results indicate that the high expression level of CBE adversely affects multiplex genome editing.

For genetic dereplication through the knockout of the known antibiotic biosynthetic genes from strains producing multiple antibiotics, an efficient and rapid genome editing tool is required. Although multiplex genome editing has been reported using CRISPR-Cas in various microorganisms, the editing efficiency drastically decreases as the simultaneous editing sites increased (Adiego-Pérez et al., 2019). Such multiplex genome editing has rarely been performed on more than three targets due to the cellular burden caused by multiple DSBs and the need for homologous recombination (Adiego-Pérez et al., 2019; Liu et al., 2019). On the other hand, CBE is expected to have significant multiplexing capacity as it does not have the limitations mentioned above (Komor et al., 2016). The potential of CBE-mediated multiplex genome editing has been reported previously (Banno et al., 2018; Wang et al., 2018; Tong et al., 2019), but its efficiency with an increasing target number needs to be addressed. Here, we showed that highly efficient multiplex editing was possible by replacing the strong promoter P*_grac_* with a weak promoter P*_spac_*. Therefore, our findings indicate that it is important to appropriately regulate the expression of base editors during multiplex genome editing. The CBE4 was shown to simultaneously edit double, triple, quadruple, and quintuple genes with 100, 100, 83, and 75% efficiency, respectively. Contrary to the previous studies, our study demonstrated that more than three targets could be edited simultaneously with a very high efficiency. The slightly reduced efficiencies in the simultaneous editing of quadruple and quintuple targets were due to a partial loss of sgRNA cassettes rather than plasmid loss. These issues may be overcome by using gRNA polycistronic cassettes (Adiego-Pérez et al., 2019), which can further increase the efficiency with the increasing number of targets.

Since CBEs can change bases at the unwanted off-target sites, it is important to consider off-target mutations in base editor-mediated genome engineering. In the genome-wide off-target mutational analysis, C-to-T or G-to-A conversion accounted for the majority of SNVs, which meant that CBE supported apparent off-target mutations, as reported previously (Liang and Huang, 2019). The off-target mutations occurred at the gRNA-independent site, and there were no common off-target sites between the multiplex and single edited strains, suggesting that DNA deamination was randomly induced without the guidance of gRNA-dCas9. Moreover, off-target SNVs did not accumulate even when the number of gRNAs increased. These results suggest that the duration or number of CBE treatments, and not the number of targets, will have a large impact on the off-target mutations. Therefore, we recommended that one-step editing should be performed rather than iterative editing to minimize the off-target effects during CBE-mediated multiplex genome editing. Using an in-depth analysis of the sequence context of the 76 SNVs in the off-target sites, we found that ABC:GVT (mutable positions underlined; “B” indicates C, G, or T; “V” indicates G, C, or A), which is known as a mutable motif of PmCDA1 (Lada et al., 2011), accounted for 51.3% (39/76) of the SNVs. Additionally, the TC:GA motif was present in 72.3% (55/76) of the SNVs, suggesting the existence of a mutable hotspot motif of PmCDA1.

Recent studies have found previously untapped or hidden microbial sources to discover potential new antibiotics (Genilloud, 2019). Actinobacteria, especially streptomycetes, have been the most well-studied as a source of new antibiotics, and a new class of antibiotics has recently been discovered in other species (Phillips et al., 2011; Piddock, 2015; Chevrette et al., 2019). Among the many strains that remain under-explored, Bacillales have prolific potential to produce structurally diverse secondary metabolites (Sumi et al., 2015; Zhao and Kuipers, 2016; Grubbs et al., 2017). Extensive investigations of the whole genome sequence of Bacillales revealed that *Bacillus*, *Paenibacillus*, and *Geobacillus* are good producers of ribosomally synthesized peptides, NRPs, and PKs (Zhao and Kuipers, 2016). However, despite the great potential of these microbes to serve as sources of new antibiotics, the biggest hurdle has been the existence of the enormous background of known compounds (Lewis, 2013). To remove known antibiotics, dereplication tools based on analytical methods have been developed, but starting from microbial extracts is a laborious process, which makes initial screening and subsequent research difficult (Gaudêncio and Pereira, 2015). Cox et al. (2017) have developed an antibiotic resistance platform (ARP) that uses indicator strains exhibiting antibiotic resistance for antibiotic dereplication. However, efficient and rapid methods for direct dereplication in original strains have not been yet reported. Genetic dereplication of known antibiotics by inactivation of the biosynthetic genes from the bacterial genome can make the activity-based screening, separation, and purification processes efficient, and be highly useful in subsequent studies, such as those on the activation of cryptic BGCs, metabolic engineering, and novel bioengineered derivatives. In this study, we evaluated the capacity of CBE as an antibiotic dereplication tool. Some mutants edited by CBE4 might have off-target mutations that affected the production of target antibiotics, but depending on the off-target mutation frequency, they probably constituted a very small fraction of the total transformants. Moreover, speed is important in genome-based antibiotic discovery because of the need to cover a large number of strains. Therefore, the CBE system may serve as a powerful tool for rapid genetic dereplication, which will greatly accelerate genome-based antibiotic discovery.

In summary, the CBE system developed in this study was shown to be highly efficient for simultaneous editing of multiple targets in *B. subtilis*, and was first applied for multi-target editing in *P. polymyxa* E681. If appropriate broad host range vectors are used, CBE4 can be applied to a wide variety of Bacillales species, beyond *Bacillus* and *Paenibacillus* species, and can be effectively used in fields such as phenotypic studies and metabolic engineering. In the future, CBE4 would be further developed to have a higher fidelity and editing capacity by reducing the off-target effects (Doman et al., 2020) and increasing the target range (Kim et al., 2017).

## Data Availability Statement

The datasets presented in this study can be found in online repositories. The names of the repository/repositories and accession number(s) can be found in the article/Supplementary Material.

## Author Contributions

MSK and S-KC designed the experiment and wrote the paper. MSK, H-RK, and D-EJ realized all experiments. All authors reviewed the manuscript. All authors contributed to the article and approved the submitted version.

### Conflict of Interest

The authors declare that the research was conducted in the absence of any commercial or financial relationships that could be construed as a potential conflict of interest.

## Footnotes

^1^https://crispy.secondarymetabolites.org/
