# Supplementary material for: Cytosine Base Editor-Mediated Multiplex Genome Editing to Accelerate Discovery of Novel Antibiotics in Bacillus subtilis and Paenibacillus polymyxa
Source: Front Microbiol. 2021 May 28;12:691839. doi: 10.3389/fmicb.2021.691839 (PMC8193733; doi:10.3389/fmicb.2021.691839)
Supplement: Supplementary file 2 [file Table_2.DOCX]

**Supplementary Material**

The following Supplementary Material is available for this article:

**Supplementary Figure S1**. Transformation efficiency of Cas9 variants and sequencing analyses of CBE-edited mutants.

**Supplementary Figure S2.** Simultaneous double and triple gene editing using P*_grac_* promoter to express CBE4.

**Supplementary Figure S3.** Antibacterial and antifungal activities of *P. polymyxa* strains.

**Supplementary Figure S4.** Purification and structural analysis of polyketides.

**Supplementary Table S1.** Bacterial strains used in this study.

**Supplementary Table S2.** Plasmids used in this study.

**Supplementary Table S3.** Oligonucleotides and primers used in this study

**Supplementary Table S4.** DNA sequences for CBE vector components

**Supplementary References**


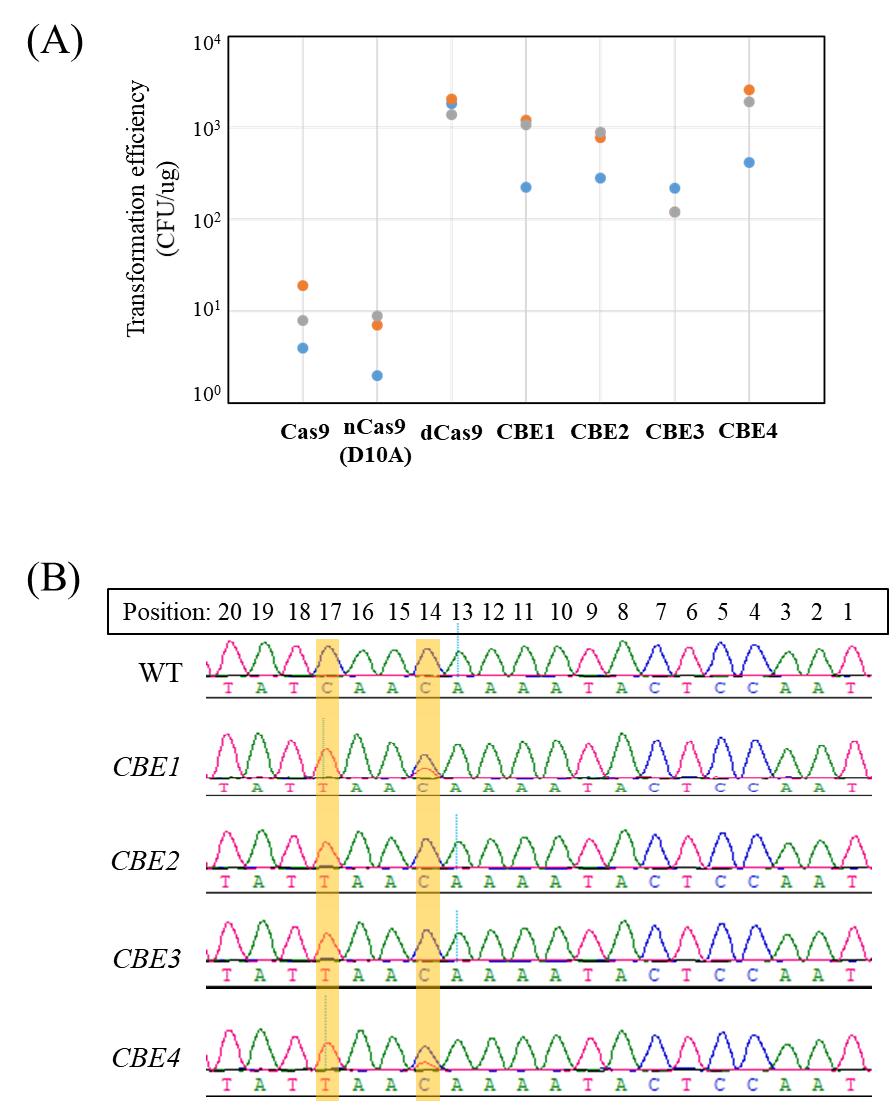


**Supplementary Figure S1.** (**A**) Transformation efficiency of the plasmids harboring one of each Cas9 variants (Cas9, nCas9, dCas9, CBE1, CBE2, CBE3, and CBE4) with sgRNA targeting the *amyE* gene. Dots represent three independent experiments. (**B**) Sequencing analyses of GFP-negative mutants edited by cytosine base editors (CBEs). The area marked with a yellow shade is the location of the expected mutation.


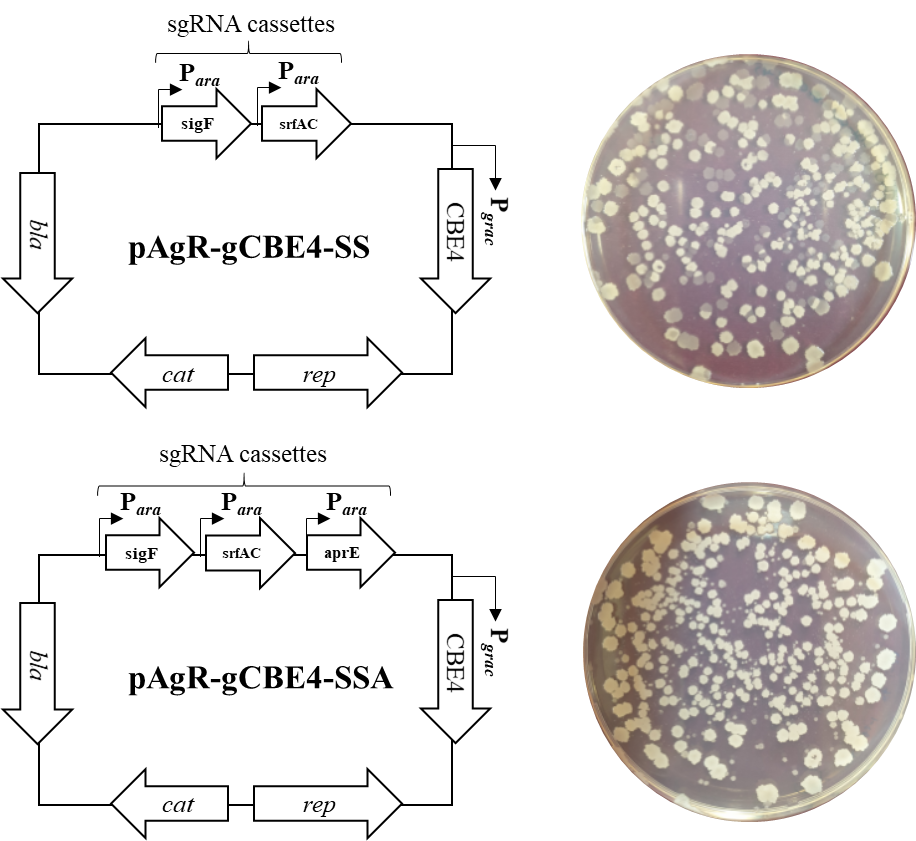


**Supplementary Figure S2. S**imultaneous double and triple gene editing using P*_grac_* promoter to express CBE4. About 50% of the transformants for double targets showed *sigF* mutants with a transparent morphology. However, the *sigF* mutation phenotype was not observed in transformants for triple targets.


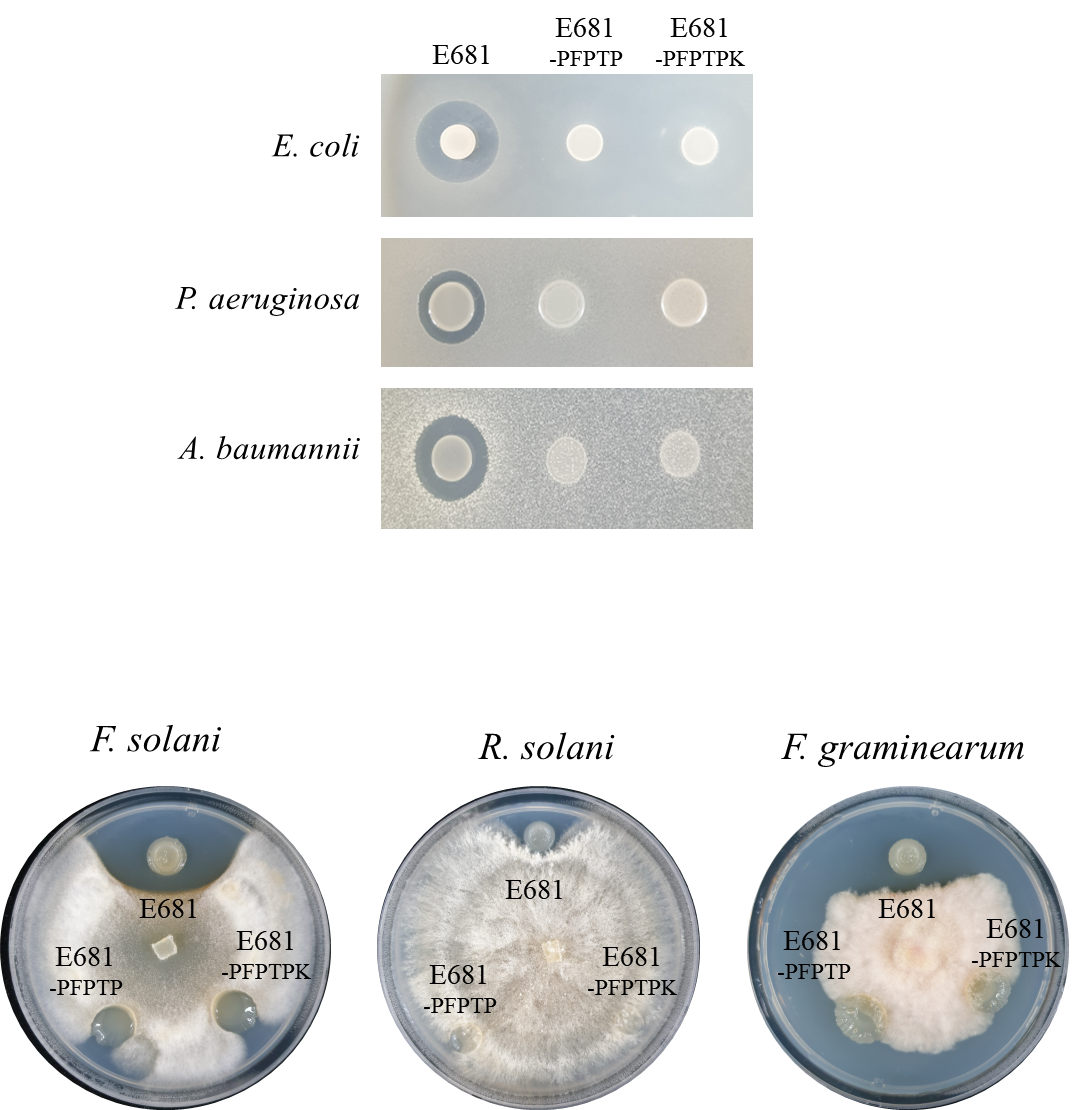


**Supplementary Figure S3.** Antibacterial and antifungal activities of *P. polymyxa* strains.


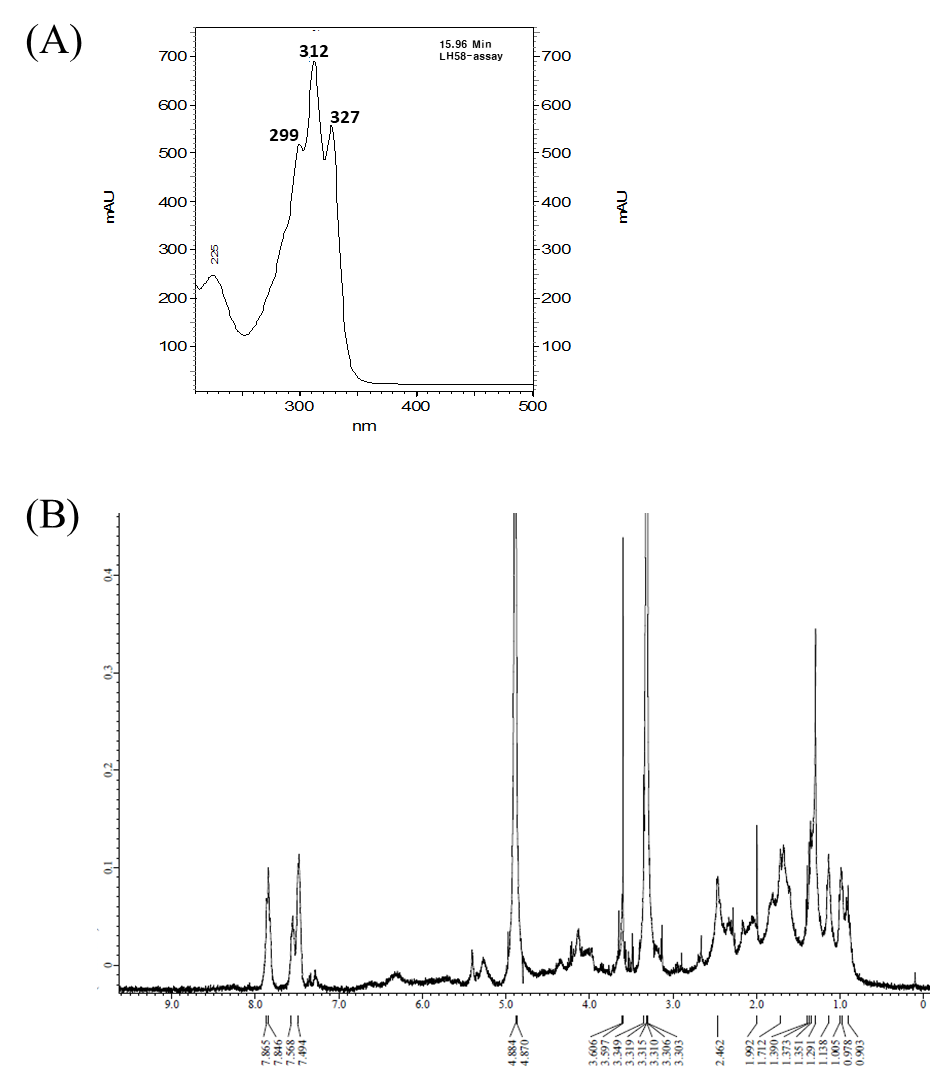


**Supplementary Figure S4.** Purification and structural analysis of polyketides. **(A)** UV spectrum of polyketide. The separation and purification of polyketides and the high-performance liquid chromatography (HPLC) analysis were performed at NPchem (Iksan-si, Jeollabuk-do, Republic of Korea). The culture broth was partitioned using ethyl acetate, and the ethyl acetate layer was purified via chromatography by using the Diaion HP-20 column (washed by 40% aq. MeOH and eluted by MeOH) and an octadecyl silica (ODS) column (washed by 40% aq. MeOH and eluted by 50% aq. MeOH), followed by Sephadex LH-20 column chromatography eluted with 70% aq. MeOH. Active fractions were analyzed by HPLC (Hitachi, Tokyo, Japan) equipped with a photodiode array detector and an ODS column (TSK-GELODS-100V; TOSOH, Tokyo, Japan). The active fractions showing antimicrobial activity against *M. luteus* were pooled and lyophilized to obtain 2 mg of dried powder. **(B)** ^1^H NMR spectrum of polyketides. For structural analysis, ^1^H NMR spectrum was obtained using JNM-ECA600 600MHz FT-NMR spectrometer (JEOL, Tokyo, Japan) in CD_3_OD. The peak was observed to be very broad overall, so it was estimated that the chemical structure could not be interpreted.

**Supplementary Table S1.** Bacterial strains used in this study.

| **Strain** | **Genotype** | **Reference** |
| --- | --- | --- |
| ***Escherichia coli*** |  |  |
| DH5α | F′ Φ80*lacZ*△M15 △(*lacZYA*-*argF*)U169 *deoR* *recA1* *endA1* *hsdR17*(rk^-^, mk^+^) *phoA* *supE*44 *thi*-1 *gyrA*96 *relA1* | Enzynomics |
| MC1061 | *araD*139 △(*araA*-*leu*)7697 △(*lac*)X74 *galK*16 *galE*15(*GalS*) lambda- e14- *mcrA0* *relA1* *rpsL*150(strR) *spoT1* *mcrB1* hsdR2 | Laboratory stock |
| S17-1 | *recA* *pro* *hsdR* RP4-2-Tc::Mu-Km::Tn7 integrated into the chromosome | KCTC 2432 |
| ***Bacillus subtilis*** |  |  |
| 168 | *trpC2* | Laboratory stock |
| BS168-gfp | BS168 *amyE*::P*_cyr3Aa_-gfp* | Jeong, et al., 2018 |
| BS5417 | BS168 *thrC*::P*_xyl_-comK* | Jeong, et al., 2015 |
| BS-A | BS5417 △*aprE* | This study |
| BS-N | BS5417 △*nprE* | This study |
| BS-W | BS5417 △*wprA* | This study |
| BS-S | BS5417 △*srfAC* | This study |
| BS-F | BS5417 △*sigF* | This study |
| BS-AN | BS5417 △*aprE* △*nprE* | This study |
| BS-ANW | BS5417 △*aprE* △*nprE* △*wprA* | This study |
| BS-ANWS | BS5417 △*aprE* △*nprE* △*wprA* △*srfAC* | This study |
| BS-ANWSF | BS5417 △*aprE* △*nprE* △*wprA* △*srfAC* △*sigF* | This study |
| ***Paenibacillus polymyxa*** |  |  |
| E681 | Wild type | Laboratory stock |
| E681-PFPTP | E681 △*pnlB* △*fusA* △*phnC* △*triD* △*pmxE* | This study |
| E681-PFPTPK | E681 △*pnlB* △*fusA* △*phnC* △*triD* △*pmxE* △*PPE_03011* | This study |

**Supplementary Table S2.** Plasmids used in this study.

| **Plasmid** | | **Description** | **Reference** |
| --- | --- | --- | --- |
| pUC19 | For cloning of CBE plasmids | | Laboratory stock |
| pUC-*dCas9* | pUC19 containing *dCas9* | | This study |
| pScI_*dCas9-CDA-UL* | Plasmid containing components of *pmCDA1* and *UGI* | | Addgene plasmid # 108551 |
| pUC-*CBE1* | pUC19 containing *CBE1* | | This study |
| pUC-*CBE2* | pUC19 containing *CBE2* | | This study |
| pUC-*CBE3* | pUC19 containing *CBE3* | | This study |
| pUC-*CBE4* | pUC19 containing *CBE4* | | This study |
| pAD123 | *E.coli* -*Bacillus* shuttle vector | | Bacillus Genetic Stock Center |
| pAgR | Plasmid pAD123 derivative containing synthetic sgRNA  module | | So, et al., 2017 |
| pAgR-P*_grac_* | pAgR containing P*_grac_* | | This study |
| pAgR-P*_spac_* | pAgR containing P*_spac_* | | This study |
| pAgR-*gCBE1* | pAgR with *CBE1* under P*_grac_* | | This study |
| pAgR-*gCBE2* | pAgR with *CBE2* under P*_grac_* | | This study |
| pAgR-*gCBE3* | pAgR with *CBE3* under P*_grac_* | | This study |
| pAgR-*gCBE4* | pAgR with *CBE4* under P*_grac_* | | This study |
| pAgR-*sCBE4* | pAgR with *CBE4* under P*_spac_* | | This study |
| pMgR-P*_spac_* | pAgR-P*_spac_* with removal of BsaI sites and repeated region within plasmid | | This study |
| pMGold-*sCBE4* | Based on pMgR-P*_spac_*, sgRNA scaffold replaced with Golden-Gate assembly site, *CBE4* insert | | This study |
| pAgR-*gCBE1*-*gfp* | pAgR-*gCBE1* with sgRNA:*gfp* | | This study |
| pAgR-*gCBE2*-*gfp* | pAgR-*gCBE2* with sgRNA:*gfp* | | This study |
| pAgR-*gCBE3*-*gfp* | pAgR-*gCBE3* with sgRNA:*gfp* | | This study |
| pAgR-*gCBE4*-*gfp* | pAgR-*gCBE4* with sgRNA:*gfp* | | This study |
| pAgR-*gCBE4*-*amyE*-g1 | pAgR-*gCBE4* with sgRNA:*amyE*-g1 | | This study |
| pAgR-*gCBE4*-*amyE*-g2 | pAgR-*gCBE4* with sgRNA:*amyE*-g2 | | This study |
| pAgR-*gCBE4*-*amyE*-g3 | pAgR-*gCBE4* with sgRNA:*amyE*-g3 | | This study |
| pAgR-*gCBE4*-*amyE*-g4 | pAgR-*gCBE4* with sgRNA:*amyE*-g4 | | This study |
| pAgR-*gCBE4*-*amyE*-g5 | pAgR-*gCBE4* with sgRNA:*amyE*-g5 | | This study |
| pAgR-*gCBE4*-*amyE*-g6 | pAgR-*gCBE4* with sgRNA:*amyE*-g6 | | This study |
| pAgR-*gCBE4*-SS | pAgR-*gCBE4* with sgRNA:*sigF*-stop and *srfAC*-stop | | This study |
| pAgR-*gCBE4*-SSA | pAgR-*gCBE4* with sgRNA:*sigF*-stop, *srfAC*-stop, and *aprE*-stop | | This study |
| pAgR-*sCBE4*-A | pAgR-*sCBE4* with sgRNA:*aprE*-stop | | This study |
| pAgR-*sCBE4*-N | pAgR-*sCBE4* with sgRNA:*nprE*-stop | | This study |
| pAgR-*sCBE4*-W | pAgR-*sCBE4* with sgRNA:*wprA*-stop | | This study |
| pAgR-*sCBE4*-S | pAgR-*sCBE4* with sgRNA:*srfAC*-stop2 | | This study |
| pAgR-*sCBE4*-F | pAgR-*sCBE4* with sgRNA:*sigF*-stop2 | | This study |
| pAgR-*sCBE4*-AN | pAgR-*sCBE4* with sgRNA:*aprE*-stop and *nprE*-stop | | This study |
| pAgR-*sCBE4*-ANW | pAgR-*sCBE4* with sgRNA:*aprE*-stop, *nprE*-stop, and *wprA*-stop | | This study |
| pAgR-*sCBE4*-ANWS | pAgR-*sCBE4* with sgRNA:*aprE*-stop, *nprE*-stop, *wprA*-stop, and *srfAC*-stop2 | | This study |
| pAgR-*sCBE4*-ANWSF | pAgR-*sCBE4* with sgRNA:*aprE*-stop, *nprE*-stop, *wprA*-stop, *srfAC*-stop2, and *sigF*-stop2 | | This study |
| pMGold-*sCBE4*-PFPTP | pMGold-*sCBE4* with sgRNA: *pnlB*, *fusA*, *phnC*, *triD*, and *pmxE* | | This study |
| pAgR-*sCBE4*-K | pAgR-*sCBE4* with sgRNA:*PKS*-3 | | This study |

**Supplementary Table S3.** Oligonucleotides and primers used in this study

| **Oligonucleotide** | **Sequence (5’ to 3’)** | **Purpose** |
| --- | --- | --- |
| *gfp*-sgRNA-BE-F | ATTG**TATCAACAAAATACTCCAAT** | Oligonucleotides for cloning of synthetic gRNA sequence used in this study |
| *gfp*-sgRNA-BE-B | AAAC**ATTGGAGTATTTTGTTGATA** |  |
| *amyE*-g1-F | ATTG**CGCCTATTTGGCTTTTCCCC** |  |
| *amyE* -g1-B | AAAC**GGGGAAAAGCCAAATAGGCG** |  |
| *amyE* -g2-F | ATTG**TCAGGTCTGGAAAAGAAAAG** |  |
| *amyE* -g2-B | AAAC**CTTTTCTTTTCCAGACCTGA** |  |
| *amyE* -g3-F | ATTG**TTCTTTTCCAGACCTGAGGG** |  |
| *amyE* -g3-B | AAAC**CCCTCAGGTCTGGAAAAGAA** |  |
| *amyE* -g4-F | ATTG**GATCAATTCACAATCGGAAA** |  |
| *amyE* -g4-B | AAAC**TTTCCGATTGTGAATTGATC** |  |
| *amyE* -g5-F | ATTG**CACTCCCGCGATCGCCTATT** |  |
| *amyE* -g5-B | AAAC**AATAGGCGATCGCGGGAGTG** |  |
| *amyE* -g6-F | ATTG**ATCCTCTGTCTCTATCAATA** |  |
| *amyE* -g6-B | AAAC**TATTGATAGAGACAGAGGAT** |  |
| *aprE*-stop-F | ATTG**TACCAGGACGGCAGTTCTCA** |  |
| *aprE*-stop-B | AAAC**TGAGAACTGCCGTCCTGGTA** |  |
| *nprE*-stop-F | ATTG**CTCAATACAATAACGCTGCA** |  |
| *nprE*-stop-B | AAAC**TGCAGCGTTATTGTATTGAG** |  |
| *wprA*-stop-F | ATTG**ACAGCCGTCAGATTTAGAAG** |  |
| *wprA*-stop-B | AAAC**CTTCTAAATCTGACGGCTGT** |  |
| *srfAC*-stop2-F | ATTG**CCAGCTCCAGCCATTCGGGG** |  |
| *srfAC*-stop2-B | AAAC**CCCCGAATGGCTGGAGCTGG** |  |
| *sigF*-stop2-F | ATTG**GCAGGAGATCGCTGACCATT** |  |
| *sigF*-stop2-B | AAAC**AATGGTCAGCGATCTCCTGC** |  |
| *pnlB*-F | ATTG**TCGAATGACGTTTGAAGCTG** |  |
| *pnlB*-B | AAAC**CAGCTTCAAACGTCATTCGA** |  |
| *fusA*-F | ATTG**ACAATCCGGCGAGGCTTATT** |  |
| *fusA*-B | AAAC**AATAAGCCTCGCCGGATTGT** |  |
| *pmxE*-F | ATTG**GAACAGCTGGCCCAAAGAAT** |  |
| *pmxE*-B | AAAC**ATTCTTTGGGCCAGCTGTTC** |  |
| *phnC*-F | ATTG**CAGCAAAACCAGTTAGAGGC** |  |
| *phnC*-B | AAAC**GCCTCTAACTGGTTTTGCTG** |  |
| *triD*-F | ATTG**CCAAGAATTGCCGGGTTACA** |  |
| *triD*-B | AAAC**TGTAACCCGGCAATTCTTGG** |  |
| *PKS*-3-F | ATTG**ACAAGCGCTTTGGGGTCATA** |  |
| *PKS*-3-B | AAAC**TATGACCCCAAAGCGCTTGT** |  |
| RBS-F | GGCCGATTAACTAATAAGGAGGACAAACATG | For RBS insertion |
| RBS-B | TATCCATGTTTGTCCTCCTTATTAGTTAATC |  |
| **Primer** | **Sequence (5’ to 3’)** | **Purpose** |
| *lacI*-*grac*-F | GAGACGATGCCAAAGAGCTCAGATCTGAGCTCAGGCCTTAACTCAC | Primers for construction of the pAgR-P*_grac_* and pAgR-P*_spac_* |
| *lacI*-*grac*-R | AGACTAGTTTTTGGCCGGCCGGGAATTGTTATCCGCTCAC |  |
| *lacI*-*spac*-R | AGACTAGTTTTTGGCCGGCCCTTAATTGTTATCCGCTCACAA |  |
| ter-F | GGCCGGCCAAAAACTAGTCTCGAGTAAGGATCTCCAGG |  |
| ter-R | AAGGGATGCAGTTTATGCATTGTACACCATGGACGCGTGACGTGAA |  |
| *Cas9*-F | AAACGACGGCCAGTGAATTCGGCCGGCCAAAAGCAGGTGAAACACCTGCAATGGATAAGAAATACTCAATAGGC |  |
| *Cas9*-R | ACCATGATTACGCCAAGCTTCTCCTCGAGGAGACTAGTTAGTCACCTCCTAGCTGAC |  |
| *CDA*-UL-F | TGAGTCAGCTAGGAGGTGACGGTGGAGGAGGTTCTGGAGG | Primers for construction of the pUC-*CBE* plasmids |
| *CDA*-UL-R | CAGCTATGACCATGATTACGACTAGTTTATGCAACCAGTCCTAGCA |  |
| *CDA*-UL-R2 | CAGCTATGACCATGATTACGACTAGTTTATAGCATCTTGATCTTGTTCTCTC |  |
| *APOBEC*-F | GCCAGTGAATTCGGCCGGCCGATTAACTAATAAGGAGGACAAACATGAGCTCAGAAACGGGCCC |  |
| *APOBEC*-R | CCTATTGAGTATTTCTTATCAGAACCTCCGCTAGACCCAC |  |
| *UGI*-F | TGAGTCAGCTAGGAGGTGACTCTGGTGGTTCTGGAGGATCTGGTGGTTCTACCAACCTTTCCGACATCAT |  |
| *UGI*-R | CAGCTATGACCATGATTACGACTAGTTTATAGCATCTTGATCTTGTTCTCTC |  |
| *UGI*-R2 | AGAACCACCAGATCCTCCAGAACCACCAGATAGCATCTTGATCTTGTTCTCTC |  |
| *UGI*-F2 | CTGGAGGATCTGGTGGTTCTACCAACCTTTCCGACATCAT |  |
| mpAD-F1 | AGTGCTGCAATGATACCGCGGGACCCACGCTCACCGGCTCC | Primers for construction of the multiplexed *CBE* plasmid |
| mpAD-R1 | ATGGTTTCTTAGCGATTCAC |  |
| mpAD-F2 | GTGAATCGCTAAGAAACCATGAGTGCACCATACAAAACATATTTC |  |
| mpAD-R2 | TCCGGAGACGGGCAAGTTAAA |  |
| mpAD-F3 | TTTAACTTGCCCGTCTCCGGATCTCTCTTAAACGAAAGATACTC |  |
| mpAD-R3 | CGGAATAATAGAAAGAGAAAAAGC |  |
| mpAD-F4 | TTTTCTCTTTCTATTATTCCGTGGACTTCATTTACTGGGTTTA |  |
| mpAD-R4 | CCGCGGTATCATTGCAGCACTG |  |
| *Egfp*-BsaI-F | CCTTTCGTCTTCAAGAATTCATTGGGAGACCAAAGGAGGTAAGGATCACTAG |  |
| *Egfp*-BsaI-R | AGAGTCGACACTCTGGATCCAAACGGAGACCGGGAAGACGTACGTTATTTG |  |
| BglII-sgRNA-F | GGAAGATCTAAAGATTGACAGTATAATAGTCAA | Primers for multiplex gRNA cloning |
| BamClaHin-sgRNA-R | CCGGAAGCTTATCGATGGATCCAAAAAAAGCACCGACTCGGTG |  |
| BB-vec-sgF | GGAAGATCTGGTCTCCATTGAGCCAGCAAGACAGCGATAA |  |
| Bsa-sgR1 | ACGGTCTCCCCTTAAAAAAAGCACCGACTCGGTG |  |
| Bsa-sgF1 | CTGGTCTCCAAGGAGCCAGCAAGACAGCGATAA |  |
| Bsa-sgR2 | ACGGTCTCCGAGTAAAAAAAGCACCGACTCGGTG |  |
| Bsa-sgF2 | CTGGTCTCCACTCAGCCAGCAAGACAGCGATAA |  |
| Bsa-sgR3 | ACGGTCTCCTCCTAAAAAAAGCACCGACTCGGTG |  |
| Bsa-sgF3 | CTGGTCTCCAGGAAGCCAGCAAGACAGCGATAA |  |
| Bsa-sgR4 | ACGGTCTCCTGATAAAAAAAGCACCGACTCGGTG |  |
| Bsa-sgF4 | CTGGTCTCCATCA AGCCAGCAAGACAGCGATAA |  |
| SCBB-vec-sgR | GGAGTCGACATCGATGGATCCGGTCTCCAAACAAAAAAAGCACCGACTCGGTG |  |
| *gfp*-F | TGTAAAACGACGGCCAGTGA | For amplifying sequencing fragment of the CBE-edited strain |
| *gfp*-R | GTGGGCCATAATTCAATTCG |  |
| *amyE*-F | GGATATACAGCCATTCAGACA |  |
| *amyE*-R | CAGCGTGTAAATTCCGTCTG |  |
| *aprE*-F | CGGATTTAAACAGACAATGAGTG |  |
| *aprE*-R | GTAAGTGCCTCCAGGAAGTG |  |
| *sigF*-F | GCCTGAAATGGGAACAACGA |  |
| *sigF*-R | TCCTCCAAATCGCTGATCGC |  |
| *nprE*-F | CGACTGATGCCCTTGGATAC |  |
| *nprE*-R | CACGGTAATAGATTTGCTGTGA |  |
| *srfAC*-F | CACAACCGGAAAGCCAAAGG |  |
| *srfAC*-R | CTGCCGAGAACCTCAGACCA |  |
| *wprA*-F | GAAAATTCAGCTCGGTTGTG |  |
| *wprA*-B | AGGGTACTGAATATCGTTTGC |  |
| *pmxE*-F2 | GACTCCCGAACTGCCGATAC |  |
| *pmxE*-R2 | GCCGTGAATCGTCTGCTGCC |  |
| *pnlB*-F2 | TCCCGATGCGTTATTGTCAG |  |
| *pnlB*-R2 | CATAGCTTATCGGGCTCTCC |  |
| *fusA*-F2 | CAAAGTCATCTATGGGAGAG |  |
| *fusA*-R2 | CAAACAGCAACAGATGACGC |  |
| *phnC*-F2 | CTATAAAGGTCGTTTACTTCGG |  |
| *phnC*-R2 | TGAACAACACCTTAAGGACG |  |
| *PKS3*-F2 | TGAAATCAGCGGTAACTCTC |  |
| *PKS3*-R2 | CCTGAGATTTCTGGCTTGGC |  |
| *triD*-F2 | TCCCTGCGGATCATTAACGG |  |
| *triD*-R2 | CAATGCCATGTTTCCACTGA |  |
| *gfp*-seqF | ACGGGAACTACAAGACACGTG | Primers for sanger sequencing |
| *amyE*-seqF | AGAGTCGACATGGATGAGCG |  |
| *aprE*-seqF | GAGCGTTGCATATGTGGAAG |  |
| *sigF*-seqF | GATTTGTTATGGATGTGGAG |  |
| *nprE*-seqF | GATCTTTCAAAACCAACAGG |  |
| *srfC*-seqF | TGCCTCATGTCAGAAAAGCG |  |
| *wprA*-seqF | GTATCTGTATATCCAAGTAAGG |  |
| *pmxE*-seqF | TGATAAGCTTCCGCTCACGC |  |
| *pnlB*-seqF | TGGCATATGAGATTGTGTCG |  |
| *fusA*-seqF | GCCTACTATGTAGCCTCCAG |  |
| *phnC*-seqF | TATTCCCAATAAGTGCGGGC |  |
| *PKS3*-seqF | ACATGCAAGCTACTGCAATC |  |
| *triD*-seqF | TTTGGTACGCTGGAATGAGG |  |

Underlined sequences are the restriction enzyme sites.

Bolded sequences represent the 20 bp synthetic gRNA.

**Supplementary Table S4.** DNA sequences for CBE vector components

| **Component** | **Sequence** |
| --- | --- |
| *dCas9* | atggataagaaatactcaataggcttagctatcggcacaaatagcgtcggatgggcggtgatcactgatgaatataaggttccgtctaaaaagttcaaggttctgggaaatacagaccgccacagtatcaaaaaaaatcttataggggctcttttatttgacagtggagagacagcggaagcgactcgtctcaaacggacagctcgtagaaggtatacacgtcggaagaatcgtatttgttatctacaggagattttttcaaatgagatggcgaaagtagatgatagtttctttcatcgacttgaagagtcttttttggtggaagaagacaagaagcatgaacgtcatcctatttttggaaatatagtagatgaagttgcttatcatgagaaatatccaactatctatcatctgcgaaaaaaattggtagattctactgataaagcggatttgcgcttaatctatttggccttagcgcatatgattaagtttcgtggtcattttttgattgagggagatttaaatcctgataatagtgatgtggacaaactatttatccagttggtacaaacctacaatcaattatttgaagaaaaccctattaacgcaagtggagtagatgctaaagcgattctttctgcacgattgagtaaatcaagacgattagaaaatctcattgctcagctccccggtgagaagaaaaatggcttatttgggaatctcattgctttgtcattgggtttgacccctaattttaaatcaaattttgatttggcagaagatgctaaattacagctttcaaaagatacttacgatgatgatttagataatttattggcgcaaattggagatcaatatgctgatttgtttttggcagctaagaatttatcagatgctattttactttcagatatcctaagagtaaatactgaaataactaaggctcccctatcagcttcaatgattaaacgctacgatgaacatcatcaagacttgactcttttaaaagctttagttcgacaacaacttccagaaaagtataaagaaatcttttttgatcaatcaaaaaacggatatgcaggttatattgatgggggagctagccaagaagaattttataaatttatcaaaccaattttagaaaaaatggatggtactgaggaattattggtgaaactaaatcgtgaagatttgctgcgcaagcaacggacctttgacaacggctctattccccatcaaattcacttgggtgagctgcatgctattttgagaagacaagaagacttttatccatttttaaaagacaatcgtgagaagattgaaaaaatcttgacttttcgaattccttattatgttggtccattggcgcgtggcaatagtcgttttgcatggatgactcggaagtctgaagaaacaattaccccatggaattttgaagaagttgtcgataaaggtgcttcagctcaatcatttattgaacgcatgacaaactttgataaaaatcttccaaatgaaaaagtactaccaaaacatagtttgctttatgagtattttacggtttataacgaattgacaaaggtcaaatatgttactgaaggaatgcgaaaaccagcatttctttcaggtgaacagaagaaagccattgttgatttactcttcaaaacaaatcgaaaagtaaccgttaagcaattaaaagaagattatttcaaaaaaatagaatgttttgatagtgttgaaatttcaggagttgaagatagatttaatgcttcattaggtacctaccatgatttgctaaaaattattaaagataaagattttttggataatgaagaaaatgaagatatcttagaggatattgttttaacattgaccttatttgaagatagggagatgattgaggaaagacttaaaacatatgctcacctctttgatgataaggtgatgaaacagcttaaacgtcgccgttatactggttggggacgtttgtctcgaaaattgattaatggtattagggataagcaatctggcaaaacaatattagattttttgaaatcagatggttttgccaatcgcaattttatgcagctgatccatgatgatagtttgacatttaaagaagacattcaaaaagcacaagtgtctggacaaggcgatagtttacatgaacatattgcaaatttagctggtagccctgctattaaaaaaggtattttacagactgtaaaagttgttgatgaattggtcaaagtaatggggcggcataagccagaaaatatcgttattgaaatggcacgtgaaaatcagacaactcaaaagggccagaaaaattcgcgagagcgtatgaaacgaatcgaagaaggtatcaaagaattaggaagtcagattcttaaagagcatcctgttgaaaatactcaattgcaaaatgaaaagctctatctctattatctccaaaatggaagagacatgtatgtggaccaagaattagatattaatcgtttaagtgattatgatgtcgatgccattgttccacaaagtttccttaaagacgattcaatagacaataaggtcttaacgcgttctgataaaaatcgtggtaaatcggataacgttccaagtgaagaagtagtcaaaaagatgaaaaactattggagacaacttctaaacgccaagttaatcactcaacgtaagtttgataatttaacgaaagctgaacgtggaggtttgagtgaacttgataaagctggttttatcaaacgccaattggttgaaactcgccaaatcactaagcatgtggcacaaattttggatagtcgcatgaatactaaatacgatgaaaatgataaacttattcgagaggttaaagtgattaccttaaaatctaaattagtttctgacttccgaaaagatttccaattctataaagtacgtgagattaacaattaccatcatgcccatgatgcgtatctaaatgccgtcgttggaactgctttgattaagaaatatccaaaacttgaatcggagtttgtctatggtgattataaagtttatgatgttcgtaaaatgattgctaagtctgagcaagaaataggcaaagcaaccgcaaaatatttcttttactctaatatcatgaacttcttcaaaacagaaattacacttgcaaatggagagattcgcaaacgccctctaatcgaaactaatggggaaactggagaaattgtctgggataaagggcgagattttgccacagtgcgcaaagtattgtccatgccccaagtcaatattgtcaagaaaacagaagtacagacaggcggattctccaaggagtcaattttaccaaaaagaaattcggacaagcttattgctcgtaaaaaagactgggatccaaaaaaatatggtggttttgatagtccaacggtagcttattcagtcctagtggttgctaaggtggaaaaagggaaatcgaagaagttaaaatccgttaaagagttactagggatcacaattatggaaagaagttcctttgaaaaaaatccgattgactttttagaagctaaaggatataaggaagttaaaaaagacttaatcattaaactacctaaatatagtctttttgagttagaaaacggtcgtaaacggatgctggctagtgccggagaattacaaaaaggaaatgagctggctctgccaagcaaatatgtgaattttttatatttagctagtcattatgaaaagttgaagggtagtccagaagataacgaacaaaaacaattgtttgtggagcagcataagcattatttagatgagattattgagcaaatcagtgaattttctaagcgtgttattttagcagatgccaatttagataaagttcttagtgcatataacaaacatagagacaaaccaatacgtgaacaagcagaaaatattattcatttatttacgttgacgaatcttggagctcccgctgcttttaaatattttgatacaacaattgatcgtaaacgatatacgtctacaaaagaagttttagatgccactcttatccatcaatccatcactggtctttatgaaacacgcattgatttgagtcagctaggaggtgac |
| *rAPOBEC1* | atgagctcagaaacgggcccagtggcagtggaccctacattgagacggcgtatcgaaccccatgagtttgaagtattctttgatccgagagaattacgcaaggagacatgcttactttacgaaattaattgggggggacgccattccatttggagacacacgtcacaaaatactaacaaacacgttgaagtcaactttatcgaaaaattcacgacagaaagatatttctgtccgaacacacgttgttcgattacttggtttttatcctggtccccatgcggcgagtgcagtcgtgcgattacagaatttctttcgcgatatccgcatgttaccctgtttatttatatcgcaaggctgtatcatcatgctgatcctcgcaatagacagggcctgcgggatttgattagtagcggagtcacgatccaaattatgactgaacaggaatcaggatactgctggagaaactttgtcaattatagcccgtcaaatgaagcccattggccgcgctatccgcatttatgggtaagactttatgttcttgaattatattgtataatactgggcctacctccttgcttgaatattttacggaggaaacaaccacaattaacatttttcaccatcgcgcttcagtcttgtcattaccagcgattaccgcctcacatcctctgggccacaggattgaaa |
| *PmCDA1* | atgaccgacgctgagtacgtgagaatccatgagaagttggacatctacacgtttaagaaacagtttttcaacaacaaaaaatccgtgtcgcatagatgctacgttctctttgaattaaaacgacggggtgaacgtagagcgtgtttttggggctatgctgtgaataaaccacagagcgggacagaacgtggcattcacgccgaaatctttagcattagaaaagtcgaagaatacctgcgcgacaaccccggacaattcacgataaattggtactcatcctggagtccttgtgcagattgcgctgaaaagatcttagaatggtataaccaggagctgcgggggaacggccacactttgaaaatctgggcttgcaaactctattacgagaaaaatgcgaggaatcaaattgggctgtggaacctcagagataacggggttgggttgaatgtaatggtaagtgaacactaccaatgttgcaggaaaatattcatccaatcgtcgcacaatcaattgaatgagaatagatggcttgagaagactttgaagcgagctgaaaaacgacggagcgagttgtccattatgattcaggtaaaaatactccacaccactaagagtcctgctgtt |
| *UGI* | atgaccaacctttccgacatcatagagaaggaaacaggcaaacagttggtcatccaagagtcgatactcatgcttcctgaagaagttgaggaggtcattgggaataagccggaaagtgacattctcgtacacactgcgtatgatgagagcaccgatgagaacgtgatgctgctcacgtcagatgccccagagtacaaaccctgggctctggtgattcaggactctaatggagagaacaagatcaagatgcta |
| LVA tag | ctggttgca |

**Supplementary References**

Jeong, D.-E., So, Y., Park, S.-Y., Park, S.-H., and Choi, S.-K. (2018). Random knock-in expression system for high yield production of heterologous protein in *Bacillus subtilis*. *J. Biotechnol.* 266**,** 50-58. doi: 10.1016/j.jbiotec.2017.12.007.

Jeong, D.-E., Park, S.-H., Pan, J.-G., Kim, E.-J., and Choi, S.-K. (2015) Genome engineering using a synthetic gene circuit in *Bacillus subtilis*, *Nucleic Acids Res* **43**: e42

So, Y., Park, S.-Y., Park, E.-H., Park, S.-H., Kim, E.-J., Pan, J.-G., et al. (2017). A highly efficient CRISPR-Cas9-mediated large genomic deletion in *Bacillus subtilis*. *Front. Microbiol.* 8**,** 1167. doi: 10.3389/fmicb.2017.01167.
